# Investigating the Associations Between Child Autistic Symptoms, Socioeconomic Context, and Family Life: A Pilot Study

**DOI:** 10.3389/fresc.2021.748346

**Published:** 2021-12-01

**Authors:** Frank Koziarz, Caroline Roncadin, Anna Kata, Eric Duku, Amber Cauwenbergs, William Mahoney, Briano Di Rezze, Colleen Anderson, Irene Drmic, Judy Eerkes, Kathleen Dekker, Katholiki Georgiades, Lorraine Hoult, Olaf Kraus de Camargo, Olivia Ng, Peter Rosenbaum, Ronit Mesterman, Stephen J. Gentles, Sue Robertson, Teresa Bennett, Stelios Georgiades

**Affiliations:** ^1^Department of Health Research Methods, Evidence, and Impact, McMaster University, Hamilton, ON, Canada; ^2^Autism Program, McMaster Children's Hospital, Hamilton Health Sciences, Hamilton, ON, Canada; ^3^Department of Psychiatry and Behavioural Neurosciences, McMaster University, Hamilton, ON, Canada; ^4^Rehabilitation Sciences, McMaster University, Hamilton, ON, Canada; ^5^CanChild Centre for Childhood Disability Research, McMaster University, Hamilton, ON, Canada; ^6^Department of Pediatrics, McMaster University, Hamilton, ON, Canada; ^7^Department of Psychiatry and Behavioural Neurosciences, Faculty of Health Sciences, McMaster University, Hamilton, ON, Canada; ^8^Department of Community Health, Wilfrid Laurier University, Waterloo, ON, Canada

**Keywords:** autism spectrum disorder, socio-ecological framework, socio-economic context, autism symptom severity, family life

## Abstract

**Objective:** The day-to-day experience of families with an Autistic child may be shaped by both, child characteristics and available resources, which often are influenced by the socioeconomic context of the family. Using a socioecological approach, this study explored the quantitative associations between child autistic symptoms, family socioeconomic status, and family life.

**Methods:** Data came from the Pediatric Autism Research Cohort—PARC Study (pilot). Parents of children with a recent diagnosis of autism completed a set of assessments, including the Autism Family Experience Questionnaire, Autism Impact Measure, and a Sociodemographic Questionnaire. A series of multiple, iterative linear regression models were constructed to ascertain quantitative associations between child autistic symptoms, socioeconomic context, and family life.

**Results:** A total of 50 children (mean age: 76 months; SD: 9.5 months; and 84% male) with data on the variables of interest were included in the analysis. The frequency of child autistic symptoms was associated with family life outcomes (*p* = 0.02 and *R*^2^ = 24%). Once autistic symptom frequency, symptom impact, and sociodemographic variables were considered, parents of higher educational attainment reported worse family life outcomes compared to their lesser-educated counterparts. This cumulative regression model had considerable explanatory capability (*p* = 0.01, *R*^2^ = 40%).

**Conclusion:** This study demonstrates the utility of using a socioecological approach to examine the dynamic interplay between child characteristics and family circumstances. Our findings suggest that family life for parents (of an autistic child) who have obtained higher education is reported (by the parents themselves) as less satisfactory compared to that of parents without higher education, once adjusted for the autistic symptom frequency of child, symptom impact, and income. These findings can inform the design and delivery of more family-centered care pathways during the years following a diagnosis of autism.

## Introduction

Autism spectrum disorder (ASD) is a neurodevelopmental disorder characterized by impairments in reciprocal social communication and social interaction, alongside patterns of restricted, repetitive behavior (1). Autism is a complex and heterogeneous condition with variability in etiology, clinical presentation, and developmental course throughout the life span (2, 3). Considering the multifactorial presentation of autism, parents and other caregivers find themselves needing to address a wide range of concerns related to child symptoms, behaviors, day-to-day functioning, and a diverse set of caregiver and family support needs (4). In some cases, behaviors such as tantrums or sleep disturbance can lead to circumstances for the family that are quite difficult to manage (4–7). The day-to-day experience of families with a child diagnosed with autism may be shaped by access to resources, such as childcare and other support systems, which often are strongly influenced by the socioeconomic context of the family (8).

To date, several studies have examined the interplay between family context (in families with Autistic children) as it relates to finances, marital relationship, and the presence of siblings. However, most of this research has focused on the quality of life of the parents and respective relatives (9–12). Although quality of life presents a useful measure to determine the state of a physical and emotional well-being of a family, these measures often do not capture the family experience holistically. We posit that a socioecological approach may be advantageous because it considers the various interrelations among individuals and their respective immediate environments (13, 14). More specifically, this conceptual framework attempts to address the dynamic interplay between the impact that the child has on the parent, and the parent on the child. Using a socioecological approach, this study explored the quantitative associations between child autistic symptoms, family socioeconomic status, and family life.

## Materials and Methods

### Participants

A sample of 50 newly diagnosed preschool-aged children was recruited from April to December of 2019 as part of the Pediatric Autism Research Cohort—PARC Study (pilot) being conducted at McMaster Children's Hospital in Ontario, Canada. Participants were recruited into the pilot study *via* consecutive sampling who met the inclusion criteria of being <6 years of age (<6 years) at the time of ASD diagnosis. The data reported and analyzed in the current study were conducted when the cohort of children averaged 76 months of age. Families with insufficient knowledge of English to understand the consent process and complete questionnaires were not included in enrollment. After agreeing to be contacted about the study, consent forms were mailed out that contained a package of questionnaires. The consenting parents or legal guardians of the children with autism received a phone call to discuss the contents and instructions of the questionnaires. Families then mailed back signed consent forms with completed questionnaires in stamped business reply envelopes. This study was approved by the Hamilton Integrated Research Ethics Board (ID: 2902).

### Autism Family Experience Questionnaire

The Autism Family Experience Questionnaire (AFEQ) is an ecologically valid, parent-nominated measure of family experience, quality of life, and prioritized outcomes for early intervention in pediatric autism populations (15). The AFEQ is organized into four domains: *Experience of Being a Parent of a Child with Autism, Family Life, Child Development Understanding and Social Relationships*, and *Child Symptoms*. For both the total score and the domain scores, a higher score is indicative of a poorer outcome. The AFEQ includes statements that are both positively and negatively worded. To best capture the association of family context, in alignment with considerations of the socioecological model, the *Family Life* subdomain was used as the primary outcome. *Family Life*, as part of the AFEQ measure, provides a quantitative value to the holistic family experience of parents, considering they have a child diagnosed with autism. More specifically, the Family Life subdomain asks parents to quantitatively document the levels of family functioning experiences that are unique to families with a child diagnosed with ASD. The Family Life domain is operationalized in the form of a Likert scale with ranges from 1 to 5 (“always” through “never”) with some items being reversed scored. Examples of items in the Family Life domain include “*Family life is a battle,” “I feel confident to go out to family events with my child,”* “*I feel comfortable about having visitors to our home*,” and “*My child is flexible in adapting to the demands of family life*.” An example of a reverse-scored item is as follows: “I feel guilty about not giving other members of the family enough attention.”

### Autism Impact Measure

The Autism Impact Measure (AIM) is a 41-item measure that provides clinically useful information about both the frequency and the functional impact of the core symptoms of ASD (16). The measure is divided into five domains: *Repetitive Behavior, Communication, Atypical Behavior, Social Reciprocity*, and *Peer Interaction*. A higher score is indicative of a poorer outcome for each domain and the total score (i.e., the cumulative score of all domains). For each item, the frequency score is obtained using a five-point Likert scale ranging from 1 to 5 (“never” through “always”); the impact score is obtained by asking parents to rate the magnitude of the effect of each symptom on the everyday functioning of a child on a five-point Likert scale ranging from 1 to 5 (“not at all” through “severely”). For this study, the Frequency and Impact total scores (i.e., across all domains) were used to document the frequency and perceived impact that the core symptoms exerted on the family variables (i.e., AFEQ). To adjust appropriately for the heterogeneity of symptom presentation, the AIM was incorporated for analysis as a predictor variable for AFEQ: Family Life.

### Socioeconomic Context

The family socioeconomic context considered the socioeconomic status of the parents providing care for the child with ASD. Variables of total annual income and the highest education attained were included as an adapted Hollingshead index. The total annual income in households was stratified according to the following groups: low income (0–$39,999), medium income ($40,000–$89,000), and high income (≥$90,000). For educational attainment, data were organized in three levels: the “degree of high school or equivalent” was a composite outcome that included the following: no schooling, some elementary schooling, elementary schooling completed, some high school, and secondary (high) school graduation certificate or equivalent. The “degree of trade school or community college” collectively included: diploma or certificate from trade, technical, or vocational school or business college, diploma or certificate from community college, or other non-university certificate or diploma. The “degree of University or higher education” was a category comprising the following: University certificate or diploma below Bachelor's level, Bachelor's (university) degree or teacher's college, a degree in medicine, dentistry, veterinary medicine, or optometry (university certificate or diploma above bachelor level), Master's degree, and earned doctorate. The legal guardian who was responsible for filling out the questionnaire reported on their own educational attainment.

### Statistical Analysis

A series of iterative multiple linear regression analyses were performed to examine the relationship between *f* amily *life* (indexed by the AFEQ subscale), child symptom severity (indexed by the AIM Frequency and Impact scale), and the socioeconomic context of the family (annual family income and highest education level attained by the legal guardian).

The following predictor variables were determined for use in the exploratory analysis *a priori*: AIM Frequency total score, AIM Impact total score, annual income, and highest degree attained by the parent of the child with ASD.

An initial model was tested in which AFEQ Family Life was regressed onto AIM Frequency. A second model was constructed to determine associations between Family Life and AIM Impact scores once adjusted for AIM Frequency scores. A final multiple linear regression model was constructed that added annual income and highest education level as variables into the model.

There were some missing data for five items (percent missing in parentheses): AFEQ Family Life (0%), AIM Frequency (3%), AIM Impact (3%), annual income (4%), and highest education level (2%). We, therefore, conducted predictive mean matching for both continuous and categorical data, creating five multiple imputed datasets using the statistical package *mice* in RStudio. Results across the five imputed datasets demonstrated little variation; therefore, the partial *F*-tests and ANOVAs were performed with the first imputed dataset. All analyses were two-tailed with a level of significance of 0.05. Statistical analysis was performed using RStudio version 1.4.1103.

## Results

The 50 participants in this cross-sectional study had a mean age of 76 months and a SD of 9.5 months at the time of assessment. The sample composition was 84% male (*n* = 42) and 16% female (*n* = 8). Of the 50 households sampled, a total of 47 legal guardians stated they were the mother who completed the questionnaire on behalf of the child, and three represented the father of the child. From the sample, descriptive statistics on the continuous variables of AFEQ—Family Life and AIM—Impact and Frequency, and categorical variables, namely, income and education are shown in Table 1. The mean of AFEQ—Family Life, AIM Impact, and AIM Frequency was 24.2, 99.36, and 122.74, respectively. The income levels (high, medium, and low) were relatively evenly distributed amongst the parents of children in the study. Regarding education, most participants had completed trades or a community college as their highest formal educational attainment at 42% (*n* = 21).

**Table 1:** Sample descriptive statistics.

**Study variables**	**Mean**	**Minimum**	**Maximum**	**SD**
**Continuous variables**				
AFEQ—family life (9–45)	24.2	10	36	5.58
AIM—impact (41–205)	99.36	41	170	29.16
AIM—frequency (41–148)	122.74	60	165	25.51
**Categorical variables**	***N***	**%**		
**Income**				
Low	14	28%		
Medium	18	36%		
High	18	36%		
**Education**				
High school	11	22%		
Trades/community college	21	42%		
University or higher education	18	36%		
**Outcome variable**	**AFEQ: family life**			
	**Mean (SD)**		**Mean (SD)**	
Income		Education		
Low	22.9 (6.18)	High School	21.6 (5.89)	
Medium	23.4 (4.90)	Trades/Community College	24.7 (5.83)	
High	26 (5.58)	University or Higher	25.2 (4.89)	

*AFEQ, Autism Family Experience Questionnaire; AIM, Autism Impact Measure*.

In linear regression model 1, the outcome variable of Family Life was assessed by AFEQ with AIM Frequency scores regressed as the predictor variable. Statistical associations can be found in Table 2. The results have indicated that AIM Frequency had a statistically significant association with Family Life (beta = 0.11, *p* = 0.02). The understanding is that for every unit increase in AIM Frequency score, the AFEQ Family score is expected to be 0.11 units greater. In addition, the model attained an adjusted *R*^2^ of 24% with a *p*-value of <0.001 (see Table 2).

**Table 2:** Model 1: linear regression of family life and AIM frequency.

**Predictor variable**	**Beta coefficient**	**Standard error**	***t*-value**	***p*-value**
Intercept	10.52	3.41	3.09	0.03**
AIM frequency	0.11	0.03	4.10	0.02***

Adjusted R^2^ = 24%, p = 0.0001608***.

*p ≤ 0.05; **p ≤ 0.01; ***p ≤ 0.001.

In model 2, both AIM Frequency and AIM Impact were included as predictor variables and regressed onto Family Life. Only AIM Impact displayed a statistically significant association with the outcome variable (beta = 0.07, *p* = 0.04). The understanding is that for every unit increase in AIM impact, the AFEQ Family score is expected to increase by 0.07, once AIM Frequency was adjusted for in the model. However, AIM Frequency was not statistically associated with Family Life after adjusting for AIM Impact. Overall, the model exhibited an adjusted *R*^2^ of 29% with a *p*-value of <0.001. The partial *F*-statistic determined that once AIM Impact was considered within the nested model of model 1, the inclusion of AIM Impact exhibited a statistically significant increase in model fit (Partial *F*-statistic; *p* = 0.0035; see Table 3).

**Table 3:** Model 2: multiple linear regression of family life, AIM frequency, and AIM impact.

**Predictor variables**	**Beta coefficient**	**Standard error**	***t*-value**	***p*-value**
Intercept	10.08	3.30	3.10	0.04**
**Symptom severity**				
AIM frequency	0.061	0.04	1.76	0.08
AIM impact	0.07	0.03	2.17	0.04*

Adjusted R^2^ = 29%, p = <0.001***.

Partial F-Statistic (F = 4.7135, df = 1), p = 0.035*.

*p ≤ 0.05; **p ≤ 0.01; ***p ≤ 0.001.

Model 3, the final cumulative model, included the predictor variables of the previous two models (AIM Impact and AIM Frequency) but was adjusted for family socioeconomic context. Once adjusted for income and education, only the predictors of AIM Frequency and parents/guardians who had pursued higher education exhibited a statistically significant association with the outcome of *Family Life*. The beta coefficient for AIM Frequency was determined to be 0.09 with a *p*-value of 0.017, whereas the coefficient for University or higher education was 4.48 with a *p*-value of 0.01. The understanding is that for every unit increase in AIM Frequency score, the AFEQ family score is expected to increase by 0.09 once AIM Impact and education are adjusted for in the model. For higher education, the understanding is that on average participants who have obtained a higher education have on average a 4.48-point increase in Family Life score (i.e., doing worse) compared to those who attained only a high school education.

The final model attained an adjusted *R*^2^ of 40%. The remaining predictor variables were not statistically significantly associated with Family Life. The partial *F*-statistic determined that once education and income were considered in Model 2, the inclusion of socioeconomic context exhibited a statistically significant increase in model fit (Partial *F*-Statistic, *p* = 0.028), which indicates that there is sufficient association to conclude that the regression model fits the data better than the model with variables only accounting for autism symptom severity (see Table 4).

**Table 4:** Model 3: multiple linear regression of family life, AIM frequency, AIM impact, and socioeconomic context.

**Predictor variables**	**Beta coefficient**	**Standard error**	***t*-value**	***p*-value**
Intercept	5.73	3.34	1.72	0.093
**Symptom severity**				
AIM frequency	0.09	0.03	2.49	0.017*
AIM impact	0.06	0.03	2.04	0.05
**Income**				
Low income	Reference group	Reference group	Reference group	Reference group
Medium income	−2.25	1.68	−1.34	0.19
High income	1.08	1.63	0.66	0.51
**Education**				
High school	Reference group	Reference group	Reference group	Reference group
Trades/community college	1.87	1.66	1.12	0.27
University or higher education	4.48	1.71	2.62	0.01*

Adjusted R^2^ = 40%, p = < 0.001*.

Partial F-Statistic (F = 2.9949, df = 4), p = 0.028*.

*p ≤ 0.05; **p ≤ 0.01; ***p ≤ 0.001.

*AFEQ, Autism Family Experience Questionnaire; AIM, Autism Impact Measure*.

Evaluating the impact of socioeconomic context may present the issue of multicollinearity. For example, level of education and annual family income can be expected to exhibit some relationship to one another. The variance inflation factor (VIF) was used to examine the magnitude of multicollinearity between predictor variables. Because none of the predictor variables was found within the accepted range of 4–10, it can be assumed that no issues of multicollinearity impacted the model.

## Discussion

This study examined the associations between child autistic symptoms, family socioeconomic context, and family life. Using a socioecological approach and a series of multiple linear regression models, our findings demonstrate the importance of the iterative process of exploring the cumulative contribution of several factors—at both the child and family level—when trying to understand the association between symptom presence and impact, socioeconomic context, and family life. Specifically, each iteration of the regression model exhibited a considerably greater ability to explain the phenomenon under investigation. The initial model, which included only the *frequency* of core autistic symptoms, accounted for 24% of the variance explained for family life. This is an important finding which aligns with previously published research documenting similar associations between child symptom severity and parental quality of life (16–18). For example, parents of children with autism have reported experiencing a greater frequency of depressive episodes and negative emotions as the symptom severity increases (19, 20). However, it is important to acknowledge the temporal aspect of our analysis; data were collected around age 6 when the effects of symptom presentation were likely compounded with the relatively recent news of the diagnosis along with the preparation for additional and demanding responsibilities for the care of a child with autism. In the second model, where the *impact* of those symptoms was considered, 29% of the variance was explained. However, with the addition of *socioeconomic context* in the final model, adjusted for symptom frequency and impact, 40% of the variance was explained. This significant explanatory power demonstrates the utility of a socioecological approach that can offer insights into evaluating the cumulative quantitative associations among various child and family variables. Specifically, our findings highlight the importance of considering the socioeconomic context of the family above and beyond the symptom severity of a child to gain a better understanding of current family life and circumstances.

Because the evaluation of the construct of family life is closely related to other measures akin to the quality of life, it is important to assess the potential convergent validity of our findings. Studies have shown that income exhibits conflicting relationships as a predictor of quality of life and life satisfaction for parents of children diagnosed with ASD (21). In one study, the severity of ASD symptom presentation was a significant predictor of parental quality of life; however, once adjusted for family income, there was no relationship between quality of life and symptoms (22). In our current study, it was observed that once income was taken into account and adjusted for, a statistically significant relationship was observed between AIM Frequency scores and family life. By contrast, parental education level exhibited a statistically significant relationship even after adjusting for symptom frequency, symptom impact, and family income. On average, parents with higher educational attainment reported worse family life experiences than their counterparts with less education. Previous research documents effects that may provide some context for our findings. Hidalgo et al. (23) conducted an analysis of socioeconomic context on life satisfaction of parents with children with ASD and determined that mothers with a high school education or less were more likely to be satisfied with the current services and care than mothers with higher educational attainment (22, 24). The researchers speculated that parents with higher education were more aware of the diversity of autism interventions and exhibited greater concern over whether their child was receiving the optimal care. However, parents with less education were not aware of the diversity of care and thus it was speculated, for this reason, parents of higher education reported lower levels of satisfaction.

Results from the current study suggest that, compared to parents with lesser education, parents who have obtained a University degree or higher education considered their family life to be less satisfactory, once adjusted for child autistic symptom frequency, symptom, impact, and income. Previous research has suggested that raising a child with ASD can interfere with the development of a professional career of a parent, and based on that we speculate that our results may reflect parents who have attained higher education but have experienced a mismatch between their level of education and employment status and/or professional development and, as such, view their family life as less satisfying (25). An important clinical implication of our results is that service planning should be both child- and family-centered and take into account ways of achieving improved family life, such as encouraging parents to join support groups and both share their concerns and learn from experiences and perceptions of other parents.

*Strengths* of this study include the use of a socioecological approach, recruitment soon after initial diagnosis, a minimal amount of missing data, and lack of multicollinearity among variables under investigation, which may have affected the relatively small sample size. Two important *limitations* of this study are: (a) the small sample size for the number of predictors, which affects the power to detect differences between groups and (b) the cross-sectional nature of the data. An additional limitation involves the timing of data accumulation analysis. A recent diagnosis of autism for parents is a difficult and often burdening moment in their lives. Considering that the data were collected near the time of diagnosis, these findings may not generalize to parents who have begun to develop effective routines and coping strategies. Large, longitudinal mixed-method studies containing follow-up assessments of both quantitative and qualitative components are required to further explore the study findings. Conducting interviews with parents of children with ASD would better provide researchers the opportunity to understand the variability in family life between households rather than relying solely on quantitative data from a set of static questionnaires.

Using a socioecological approach and a series of multiple linear regression models, this study explained considerable variance in family life after accounting for the child's autistic symptoms and family socioeconomic context. Our results suggest that family life for parents (of an autistic child) who have obtained a University degree or higher education is considered (by parents themselves) less satisfactory compared to that of parents with less education, once adjusted for child's autistic symptom frequency, symptom impact, and income. The significant explanatory power demonstrates the utility of a socioecological approach that can offer insights into the cumulative quantitative associations among various child and family variables and family life that has clinical implications.

In line with the evidence-based care model outlined by Sackett et al., our study highlights the importance of considering family socioeconomic context when planning clinical care and allocation of service resources (26). Such an approach would inform the design and delivery of more family-centered, holistic yet pragmatic, and feasible care pathways that consider both, the characteristics of the child and the family circumstances during the years following a diagnosis of autism.

## Data Availability Statement

The raw data supporting the conclusions of this article will be made available by the authors, without undue reservation.

## Ethics Statement

The studies involving human participants were reviewed and approved by McMaster Research Ethics Board McMaster University. Written informed consent to participate in this study was provided by the participants' legal guardian/next of kin.

## Author Contributions

All authors have contributed significantly to the content of the article and have read and approve of the manuscript submission.

## Funding

This study was supported by the Faculty of Health Sciences and Department of Psychiatry and Behavioural Neurosciences at McMaster University, McMaster Children's Hospital Research Collaborative, Hamilton Health Sciences, and by an award from the Autism Spectrum Disorders Research Project of Grand Master Paul E Todd-2017-19.

## Conflict of Interest

The authors declare that the research was conducted in the absence of any commercial or financial relationships that could be construed as a potential conflict of interest.

## Publisher's Note

All claims expressed in this article are solely those of the authors and do not necessarily represent those of their affiliated organizations, or those of the publisher, the editors and the reviewers. Any product that may be evaluated in this article, or claim that may be made by its manufacturer, is not guaranteed or endorsed by the publisher.
